# Synthesis and optical property of one-dimensional spinel ZnMn_2_O_4 _nanorods

**DOI:** 10.1186/1556-276X-6-323

**Published:** 2011-04-11

**Authors:** Pan Zhang, Xinyong Li, Qidong Zhao, Shaomin Liu

**Affiliations:** 1 Key Laboratory of Industrial Ecology and Environmental Engineering and State Key Laboratory of Fine Chemical, School of Environmental Science & Technology, Dalian University of Technology, Dalian, 116024, China; 2 Department of Chemical Engineering, Curtin University, Perth, WA 6845, Australia

## Abstract

Spinel zinc manganese oxide (ZnMn_2_O_4_) nanorods were successfully prepared using the previously synthesized α-MnO_2 _nanorods by a hydrothermal method as template. The nanorods were characterized by X-ray diffraction, scanning electron microscopy, transmission electron microscopy, UV-Vis absorption, X-ray photoelectron spectroscopy, surface photovoltage spectroscopy, and Fourier transform infrared spectroscopy. The ZnMn_2_O_4 _nanorods in well-formed crystallinity and phase purity appeared with the width in 50-100 nm and the length in 1.5-2 μm. They exhibited strong absorption below 500 nm with the threshold edges around 700 nm. A significant photovoltage response in the region below 400 nm could be observed for the nanorods calcined at 650 and 800°C.

## Introduction

Spinel is an important class of mixed-metal oxides, which has the general chemical composition of AB_2_O_4_. In recent years, mixed transition-metal oxides with spinel structure have attracted much attention, owing to their various properties such as photocatalytic [1-4], electrochemical performance [5], magnetic [6,7] properties, or being used as lithium ion batteries [8]. Mn-doped ZnO has also aroused lots of interest because it had been predicted to be a room-temperature diluted magnetic semiconductor [9], which was later verified by experiments. Therefore, the Mn-Zn-O ternary systems belong to a class of interesting and useful materials in terms of their electrical and magnetic properties.

As one of the important mixed transition-metal oxides with spinel structure, ZnMn_2_O_4 _is a promising functional material and has become the focus of various researches owing to its potential applications. ZnMn_2_O_4 _could be used for the negative temperature coefficient thermistors on account of their unique electrical properties [10]. Ferrari and the coworkers studied the catalytic activity of zinc manganite for the reduction of NO by several types of hydrocarbons [4,11]. Those authors suggested that ZnMn_2_O_4 _was an efficient catalyst for the reduction of NO to N_2_, and, in all cases, its best selectivity to N_2 _and CO_2 _was at almost the maximum conversion temperature.

The physical and chemical properties of nanomaterials would be strongly affected by their particle sizes and morphologies [12]. At present, a tremendous amount of comprehensive investigations are under way into the unique applications of one-dimensional (1D) nanostructures involving nanorods, nanotubes, nanowires, and nanobelts because they provide a great opportunity to investigate the dependence of optical/electrical properties, thermal transport, and mechanical performance on the nano-scaled dimensionality and size [13,14]. 1D nanomaterials, especially nanorods have displayed enhanced performances in many fields such as catalysts [15], sensors [9], solar cells [16], and so on.

ZnMn_2_O_4 _particles could be prepared earlier by various methods, such as solid-state reaction [17,18], sol-gel [19], co-precipitation method [18], and hydrothermal method [20,21]. For instance, Bessekhouad and Trari [18] prepared spinel ZnMn_2_O_4 _powder by solid-state reaction under high temperature. Zhang et al. [21] fabricated ZnMn_2_O_4 _nanoparticles by a hydrothermal method expending 48 h. Fan et al. [22] successfully synthesized 1D single-crystalline spinel MFe_2_O_4 _nanotubes/nanorings by thermal transformation process. In this study, we prepare the ZnMn_2_O_4 _nanorods successfully using the α-MnO_2 _nanorods as templates. The ZnMn_2_O_4 _nanorods obtained thus are well characterized, demonstrating their morphology, optical, and photoelectric properties.

## Experimental

### Synthesis

All chemicals used in this study were analytic-grade reagents and used without further purification. At first, α-MnO_2 _nanorods were prepared by a hydrothermal approach. In a typical synthesis procedure, 0.8686 g KMnO_4 _and 1.8 mL concentrated HCl (37 wt%) were added to 100 mL deionized water to form the precursor solution, which was then transferred into a Teflon-lined stainless steel autoclave with a capacity of 120 mL. The autoclave was sealed and hydrothermally treated at 140°C for 12 h. After the autoclave was cooled down to room temperature naturally, the black precipitates were collected by centrifugation and washed with deionized water and anhydrous ethanol several times to remove possible impurities or excess ions. The as-prepared sample was then dried in air overnight.

To obtain ZnMn_2_O_4 _nanorods, the prepared α-MnO_2 _nanorods (0.0174 g) were placed in 20 mL distilled water at room temperature to form a mixed solution by constant stirring. A Zn(NO_3_)_2 _solution (2.5 mL, 0.1 M) was then added to the solution, which was continually stirred for 5 min. Then NaOH solution (40 mL, 0.1 M) was directly added to the above solution dropwise, and the resulting mixture was maintained for 30 min under continuous stirring. The base-treated products were washed with distilled water and anhydrous ethanol several times. Next, the products were all calcined at 500, 550, 600, 650, and 800°C for 2 h in air.

### Characterizations

The crystal structures and microstructures of the products were characterized by X-ray diffraction (XRD) (D/MAX-2400). The chemical composition of the samples was crosschecked by energy dispersive X-ray spectroscope (EDS, JSM-5600LV). The SEM (Quanta 200 FEG), transition electron microscopy (TEM)/high-resolution transition electron microscopy (HRTEM) (JEOL JEM-2000EX), and selected area electron diffraction (SAED, Tecnai G220) were employed to observe the samples morphology. UV-Vis spectrophotometer (JASCO, UV-550) and a home-built surface photovoltage (SPV) measurement system based on a lock-in amplifier (Stanford, SR830) were employed to test the optical and photoelectric properties. X-ray photoelectron spectroscopy (XPS, PHI 5600) was employed to reveal the surface chemical composition of the products. The chemical compositions of the different products were determined using Fourier transform infrared spectroscopy (FTIR, VERTEX 70).

## Results and discussion

### XRD patterns of the calcined samples

**Figure 1:**
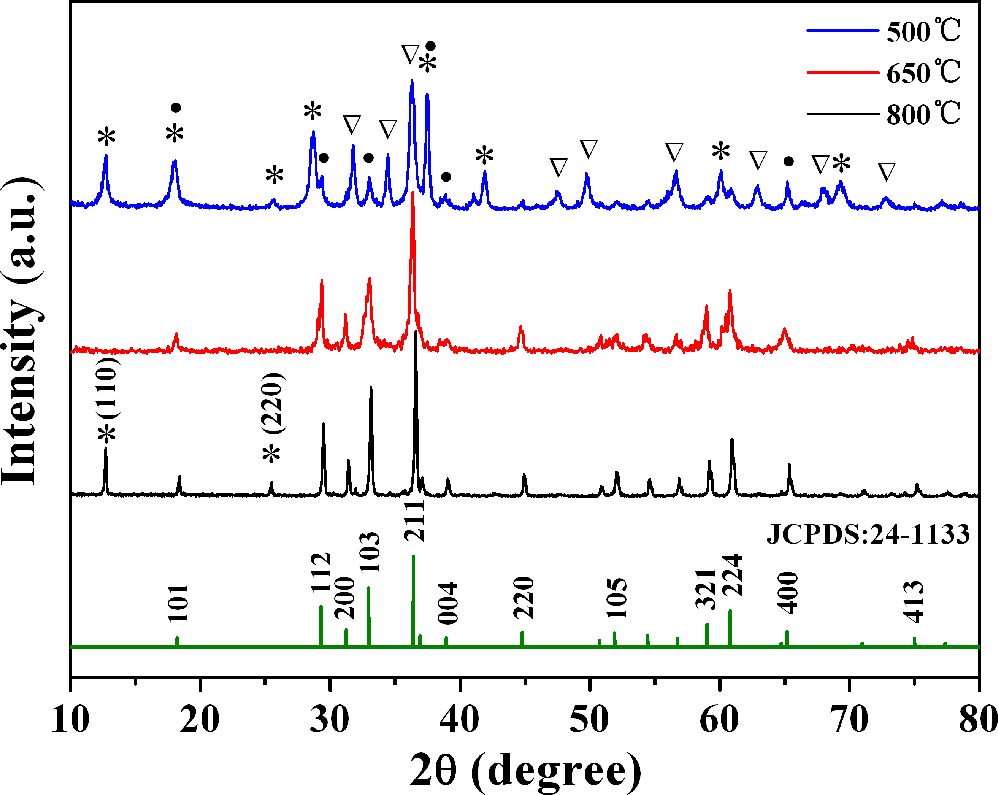
**XRD patterns of the ZnMn_2_O_4 _nanorods at different calcined temperatures and as per the standard card about ZnMn_2_O_4 _(JCPDS file No.24-1133)**. Asterisks, α-MnO_2_; inverted triangle, ZnO; filled circle, ZnMn_2_O_4_.

The crystallinities and phase purities of the ZnMn_2_O_4 _nanorods calcined were examined by powder XRD. Figure 1 shows the XRD patterns of the samples after calcination at different temperatures and as per the standard data card about the ZnMn_2_O_4_. After calcination at 650°C, all the XRD peaks of the calcined samples could be indexed to cubic ZnMn_2_O_4 _with spinel structure (JCPDS file No.24-1133), where the diffraction peaks at 2θ values of 29.3°, 33.03°, 36.3°, 59.01°, 60.77°, and 65.1° are ascribed to the reflection of (112), (103), (211), (321), (224), and (400) planes of the spinel ZnMn_2_O_4_, respectively. No peaks from other phases are detected, indicating high purity of the products. However, in the products which were calcined at 500°C, the diffraction peaks of α-MnO_2 _and ZnO could still be seen clearly, implying that the precursor was not converted into spinel ZnMn_2_O_4 _completely even at such a calcination temperature. As for the sample treated at 800°C, two peaks of α-MnO_2 _preferential growth along (110) and (220) crystal faces at the surface of ZnMn_2_O_4 _nanorods were detected again because of partial decomposition of ZnMn_2_O_4_. Meanwhile, the peaks of α-MnO_2 _and ZnO could also be seen unambiguously when the precursors were calcined at 550 and 600°C (see Figure S1 in Additional file 1). These phenomena indicate that the formation of ZnMn_2_O_4 _began at the calcination temperature of about 650°C. On increasing the calcination temperature from 500 to 800°C, the peak widths of the ZnMn_2_O_4 _nanorods become narrower, and the relative intensities of the characteristic peaks increase gradually, which implies that the spinel ZnMn_2_O_4 _crystallites were growing and transforming from the disordered to a more ordered structure [23].

### SEM images and elemental analysis of the nanorods

**Figure 2:**
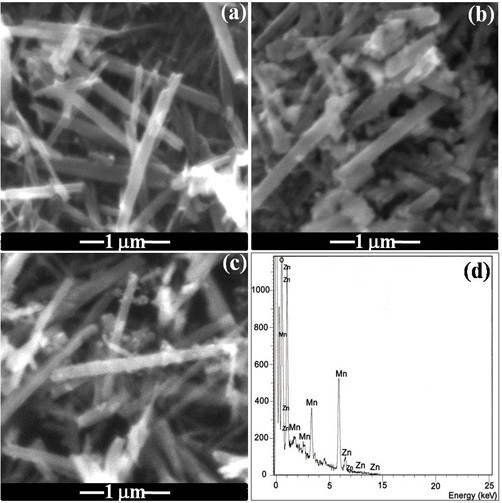
**The SEM images of different samples**: **(a) **α-MnO_2 _nanorods; **(b) **α-MnO_2_/Zn(OH)_2 _nanorods; **(c) **the ZnMn_2_O_4 _nanorods calcined at 650°C; and **(d) **EDS spectrum of the ZnMn_2_O_4 _nanorods calcined at 650°C.

The general morphology of these nanorods was observed using scanning electron microscopy (SEM). Figure 2a displays an overview image of the α-MnO_2 _nanorods. After coating Zn(OH)_2 _onto the α-MnO_2 _nanorods, the precursor of ZnMn_2_O_4 _nanorods is obtained. The SEM images in Figure 2b show that the morphology does not change with the rod width in the range of 50-100 nm and the length in the range of 1.5-2 μm. The morphology of the ZnMn_2_O_4 _obtained by calcination at 650°C of the precursor is shown in Figure 2c. It also displays a similar morphology to the MnO_2 _nanorods. The EDS characterization at the same position was carried out. As shown in Figure 2d, it is clear that the nanorods consist of Zn, Mn, and O and the elemental ratio of Zn to Mn is determined to be about 1:2.

### TEM and HRTEM images of the ZnMn_2_O_4 _nanorods

**Figure 3:**
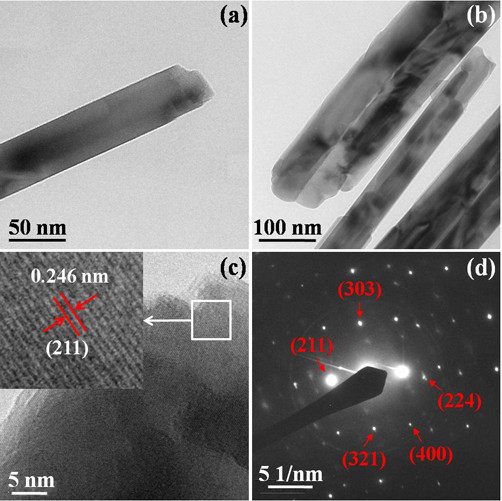
**Characterization of the ZnMn_2_O_4 _nanorods calcined at 650°C**: **(a,b) **TEM images; **(c) **HRTEM image. Inset: the enlargement of the marked square; and **(d) **SAED pattern.

The TEM and HRTEM were employed to further investigate the morphology of these nanocrystalline ZnMn_2_O_4 _superstructures calcined at 650°C (Figure 3). TEM image confirms the nanocrystalline feature of the ZnMn_2_O_4 _particles (Figure 3a,b), in which the individual rod width is in the range of 50-100 nm. Figure 3c shows the HRTEM image of ZnMn_2_O_4 _nanorods. The inset in Figure 3c is the enlargement of the selected area, indicating the good crystallization of the nanoparticles. The fringes of *d *= 0.246 nm match that of the (211) crystallographic plane of ZnMn_2_O_4_, which is the strongest crystallographic plane, corresponding to the distance between (211) crystal planes of the spinel phase of zinc manganese oxide (JCPDS file No. 24-1133). Figure 3d is the SAED image of the ZnMn_2_O_4 _nanorods, which shows the (211), (224), and (321) crystal planes clearly.

### The UV-Vis diffuse reflectance spectra of the ZnMn_2_O_4 _nanorods

**Figure 4:**
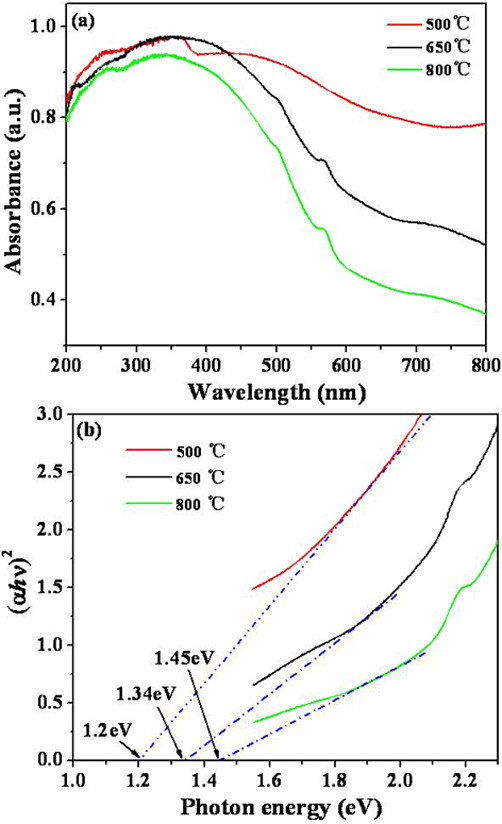
**UV-Vis spectra and plots for band gaps determination**. **(a) **The UV-Vis diffuse reflectance spectra of the ZnMn_2_O_4 _nanorods calcined at 500, 650, and 800°C, respectively; **(b) **plots of (α*hυ*)^2 ^versus photon energy of ZnMn_2_O_4 _nanorods calcined at 500, 650, and 800°C, respectively.

The optical property of the ZnMn_2_O_4 _nanorods can be observed by the UV-Vis diffuse reflectance spectroscopy. Figure 4a shows the UV-Vis diffuse reflectance spectra of the ZnMn_2_O_4 _nanorods calcined at different temperatures. All the samples have strong absorption between 300 and 500 nm, and the absorption edges of these samples are all around 700 nm. The optical band gaps could be determined from the curves of (α*hυ*)^*n *^versus *hυ*, α being the optical absorption coefficient. The exponent *n *equals 1/2 for indirectly allowed and 2 for directly allowed transitions (Figure 4). Figure 4b is the (α*hυ*)^2^-*hν *curves for the ZnMn_2_O_4 _nanorods calcined at different temperatures. The optical band gaps are calculated as 1.2, 1.34, and 1.45 eV for the samples calcined at 500, 650 and 800°C, respectively. No linear relation was found for *n *= 1/2, suggesting that the prepared ZnMn_2_O_4 _may be a semiconductor allowing direct transitions at these energy levels [3].

### XPS spectra of the ZnMn_2_O_4 _nanorods

**Figure 5:**
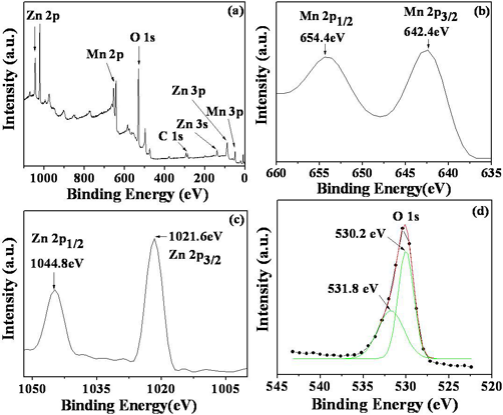
**XPS spectra of the ZnMn_2_O_4 _nanorods obtained by calcining the precursor at 650°C**: **(a) **survey of the sample; **(b) **Mn 2p; **(c) **Zn 2p; and **(d) **O 1s.

In ZnMn_2_O_4_, Mn and Zn atoms exist in the samples with more than one chemical state (A-sites or B-sites), bringing about several contributions with different binding energies in the XPS. Therefore, XPS was employed to reveal the surface chemical compositions of the nanocrystalline ZnMn_2_O_4 _superstructures obtained from the calcinations of the precursor at 650°C (Figure 5). Figure 5a shows the survey spectra of the nanocrystalline ZnMn_2_O_4_. Elements of Zn, Mn, O, and adventitious C existed in the ZnMn_2_O_4_. The carbonate species adsorbed on the surface have appeared from the calibration for XPS instrument itself. As shown in the spectrum in Figure 5b, the peaks of 654.4 and 642.4 eV can be attributed to Mn 2*p*_1/2 _and Mn 2*p*_3/2_, respectively. Figure 5c shows the Zn 2*p *peaks at binding energies of 1044.8 and 1021.6 eV. This reveals the oxidation state of Mn^3+ ^in the sample. Meanwhile, O 1s spectra of ZnMn_2_O_4 _were also recorded (Figure 5d). The broad peak of O 1s can be fitted by two peaks at binding energies of 531.8 and 530.2 eV. The stronger peak at 530.2 eV is ascribed to the characteristics of oxygen in metal oxide, and the other peak at around 531.8 eV suggests the presence of other components, such as OH, H_2_O, and carbonate species adsorbed on the surface. In addition, XPS analysis confirms that the ratio of Zn to Mn in the sample is very close to 1:2, which is in good agreement with the formula of ZnMn_2_O_4_.

### SPV spectra of the ZnMn_2_O_4 _nanorods

**Figure 6:**
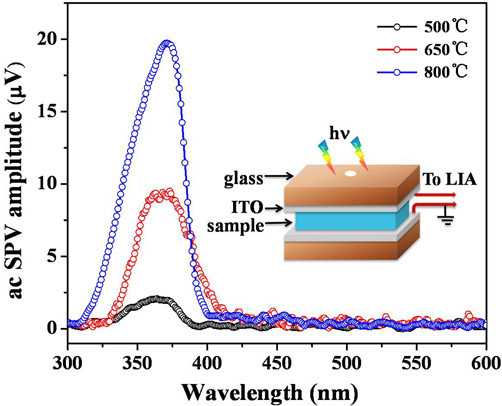
**AC SPV amplitude spectra of the ZnMn_2_O_4 _nanorods calcined at 500, 650, and 800°C**. Inset: schematic of the AC photovoltaic cells.

SPV method, a well-established technique for the characterization of photoelectric materials, can be used to investigate their photoelectric properties [24]. Figure 6 presents the obtained SPV amplitude spectra for the ZnMn_2_O_4 _nanorods calcined at 500, 650, and 800°C and the schematic of the AC photovoltaic cells. As the calcined temperature goes on increasing, the SPV response intensities become stronger and stronger because the spinel ZnMn_2_O_4 _nanorods become well ordered and the crystallinities enhance. For the ZnMn_2_O_4 _nanorods calcined at 650 and 800°C, a significant SPV response band could be observed in the region 300-400 nm, which is directly related to the free charge carriers induced by the incident light. However, only a very small response can be observed about the product calcined at 500°C because of poor crystallinity of ZnMn_2_O_4 _as well as the existence of α-MnO_2_, which almost cannot produce SPV response.

### IR spectra of the ZnMn_2_O_4 _nanorods

**Figure 7:**
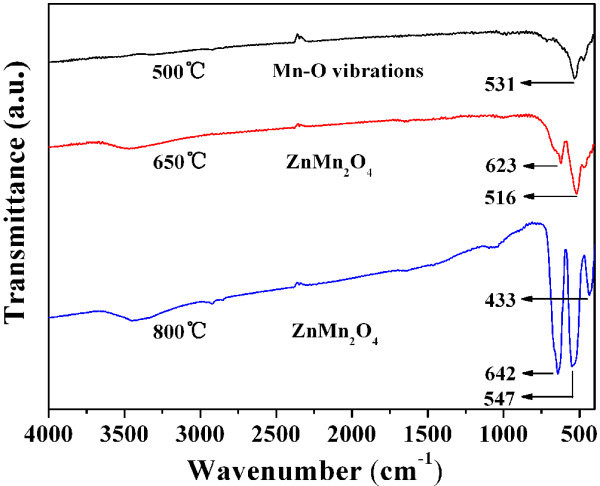
**FTIR spectra for the ZnMn_2_O_4 _nanorods calcined at 500, 650, and 800°C, respectively**.

Infrared (IR) spectroscopy could provide plentiful information on the molecular structure and chemical bonding, which enables the characterization and identification of chemical species. Herein, we employed IR spectroscopy to comparatively analyze and identify the material compositions of different products. The IR spectra of the prepared samples treated at 500, 650, and 800°C are presented in Figure 7. The spectrum of the precursor calcined at 500°C shows a band at 531 cm^-1^, which is attributed to the Mn-O vibrations of MnO_2_. The bands belonging to ZnMn_2_O_4 _nanorods are considerably weak, probably because of the co-existence of α-MnO_2 _and ZnO. When the temperature reached 650°C, a strong band at 516 cm^-1 ^and a weak one at 623 cm^-1 ^related to spinel ZnMn_2_O_4 _appear. Upon increasing the treatment temperature to 800°C, these bands become more intense and shift to higher wavenumbers, from 516 to 547 cm^-1 ^and from 623 to 642 cm^-1^, which reveals the formed spinel nanorods have achieved an improved crystallinity [25].

## Conclusions

In summary, one-dimensional spinel ZnMn_2_O_4 _nanorods were successfully fabricated using the α-MnO_2 _nanorods as template. The ZnMn_2_O_4 _nanorods mainly grew along the (211) crystalline plane with the width in 50-100 nm and the length in 1.5-2 μm. The optical band gap energies of the nanorods calcined at 500°C, 650°C, and 800°C were respectively estimated to be 1.2, 1.34, and 1.45 eV. As the calcination temperature increased, they presented with much improved crystallinity and photoelectric response. The simple method for preparing the ZnMn_2_O_4 _nanorods reported here could also be utilized to fabricate other manganates.

## Abbreviations

EDS: energy dispersive X-ray spectroscope; FTIR: Fourier transform infrared spectroscopy; HRTEM: high-resolution transition electron microscopy; SAEDL: selected area electron diffraction; SPV: surface photovoltage; TEM: transition electron microscopy; XPS: X-ray photoelectron spectroscopy; XRD: X-ray diffraction.

## Competing interests

The authors declare that they have no competing interests.

## Authors' contributions

PZ conceived of the study, carried out the experiment process, did the most parts' characterizations, and drafted the manuscript. XYL conceived of the study, guided the research, and helped in drafting the manuscript. QDZ and SML participated in research coordination, draft revision, and helped in drafting the manuscript. All the authors read and approved the final manuscript.

## Supplementary Material

Additional file 1 **Figure S1 XRD patterns of the ZnMn**_**2**_**O**_**4 **_**nanorods calcined at different temperatures**. *Asterisks, α-MnO_2_; inverted triangle, ZnO; filled circle, ZnMn_2_O_4_. Click here for file
