# Supplementary material for: Synthesis and optical property of one-dimensional spinel ZnMn2O4 nanorods
Source: Nanoscale Res Lett. 2011 Apr 11;6(1):323. doi: 10.1186/1556-276X-6-323 (PMC3211411; doi:10.1186/1556-276X-6-323)
Supplement: Additional file 1 — Figure S1 XRD patterns of the ZnMn2O4 nanorods calcined at different temperatures. *Asterisks, α-MnO2; inverted triangle, ZnO; filled circle, ZnMn2O4. [file 1556-276X-6-323-S1.DOC]

# Supporting Information for

# Synthesis and optical property of one-dimensional spinel ZnMn_2_O_4_ nanorods

### Pan Zhang^1^*, Xinyong Li ^1,2^*^§^, Qidong Zhao^1^*, Shaomin Liu^2^*^§^

^1^Key Laboratory of Industrial Ecology and Environmental Engineering and State Key Laboratory of Fine Chemical, School of Environmental Science & Technology, Dalian University of Technology, Dalian, 116024, China.

^2^ Department of Chemical Engineering, Curtin University, Perth, WA 6845, Australia

*These authors contributed equally to this work.

^§^Corresponding author

Email addresses:

PZ: zhangpanhnsd@163.com

XYL: xyli@dlut.edu.cn

QDZ: qdzhao2002@gmail.com

SML: shaomin.liu@curtin.edu.au


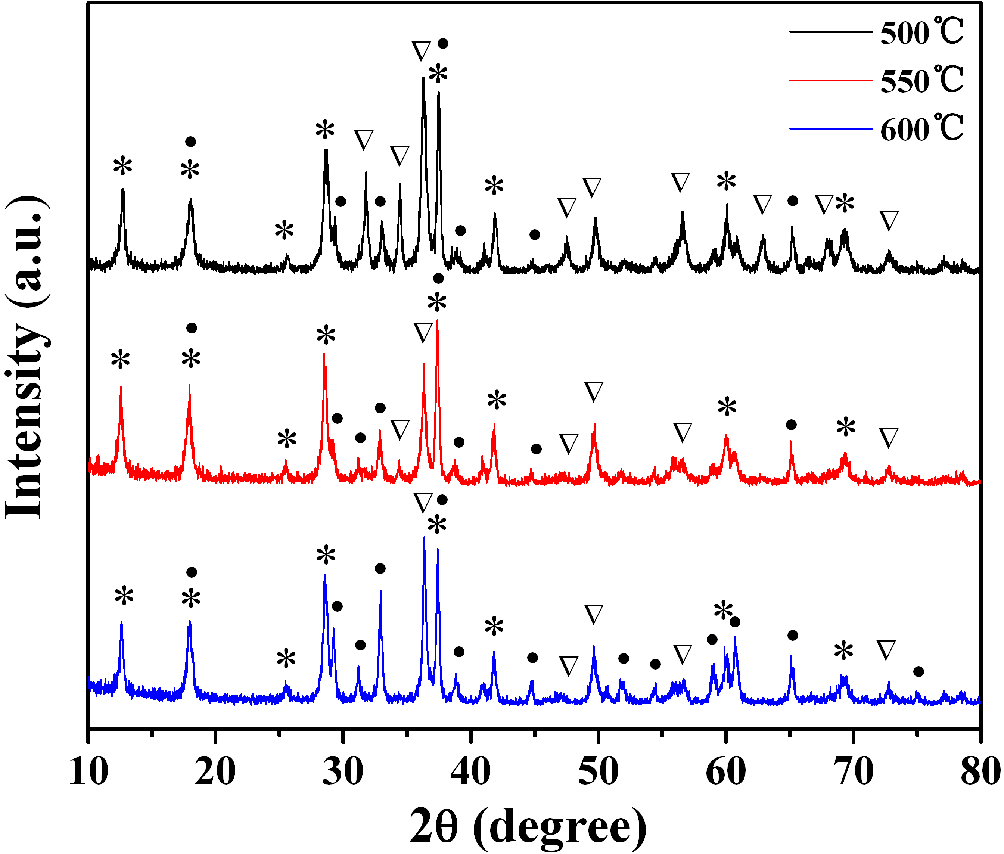


Fig. S1 XRD patterns of the ZnMn_2_O_4_ nanorods calcined at different temperatures. *: α-MnO_2_; ∇: ZnO; •: ZnMn_2_O_4_.
